# A Review on Macroscale and Microscale Cell Lysis Methods

**DOI:** 10.3390/mi8030083

**Published:** 2017-03-08

**Authors:** Mohammed Shehadul Islam, Aditya Aryasomayajula, Ponnambalam Ravi Selvaganapathy

**Affiliations:** Department of Mechanical Engineering, McMaster University, Hamilton, ON L8S 4L7, Canada; shehadul.islam@gmail.com (M.S.I.); aryasoma@mcmaster.ca (A.A.)

**Keywords:** cell lysis, cell lysis methods, microfluidics, electrical lysis, mechanical lysis, thermal lysis

## Abstract

The lysis of cells in order to extract the nucleic acids or proteins inside it is a crucial unit operation in biomolecular analysis. This paper presents a critical evaluation of the various methods that are available both in the macro and micro scale for cell lysis. Various types of cells, the structure of their membranes are discussed initially. Then, various methods that are currently used to lyse cells in the macroscale are discussed and compared. Subsequently, popular methods for micro scale cell lysis and different microfluidic devices used are detailed with their advantages and disadvantages. Finally, a comparison of different techniques used in microfluidics platform has been presented which will be helpful to select method for a particular application.

## 1. Introduction

Cell lysis or cellular disruption is a method in which the outer boundary or cell membrane is broken down or destroyed in order to release inter-cellular materials such as DNA, RNA, protein or organelles from a cell. Cell lysis is an important unit operation for molecular diagnostics of pathogens, immunoassays for point of care diagnostics, down streaming processes such as protein purification for studying protein function and structure, cancer diagnostics, drug screening, mRNA transcriptome determination and analysis of the composition of specific proteins, lipids, and nucleic acids individually or as complexes.

Based on the application, cell lysis can be classified as complete or partial. Partial cell lysis is performed in techniques such as patch clamping, which is used for drug testing and studying intracellular ionic currents [1]. In this technique, a glass micropipette is inserted into the cell, rupturing the cell membrane partially. Complete cell lysis is the full disintegration of cell membrane for analyzing DNA, RNA and subcellular components [2].

Different methods have been developed in order to lyse the cell. The nature of lysis method chosen is influenced by the ease of purification steps, the target molecules for analysis, and quality of final products [3]. Laboratory and industrial scale cell lysis methods have been developed and used for many years now. There are a few companies that have also developed equipment (e.g., sonicators and homogenizers) and chemicals (reagents, enzymes and detergents) to lyse cells, which are commercially available. The global market for cell lysis is estimated at 2.35 billion dollars in 2016 and is expected to reach 3.84 billion dollars by 2021 [4].

In past 25 years, conventional laboratory-based, manually-operated bioanalytical processes have been miniaturized and automated by exploiting the advances in microfabrication in the microelectronic industry [5] leading to emergence of a new field known as Microfluidics. Microfluidic technology involves the handling and manipulation of tiny volumes of fluids (nanoliter to picoliter) in the micrometer scale and offers various advantages which include low reagent volume, high surface to volume ratio, low cost and easy handling of small volumes of fluids which are suited for cell analysis. Microfluidic devices have shown great promise in cell lysis and in general cell analysis due to the similar operating size scale [6]. Various researchers have developed microfluidic devices to lyse cells [7]. Researchers have also developed single cell lysis techniques for single cell analysis [8]. This paper reviews several methods of cell lysis techniques that have been used in both macro and micro scale. Finally, a competitive analysis has been performed, which might be helpful to select a process to lyse cell depending on the application and motivation of lysis.

## 2. Overview of Cell Lysis

**Figure 1:**
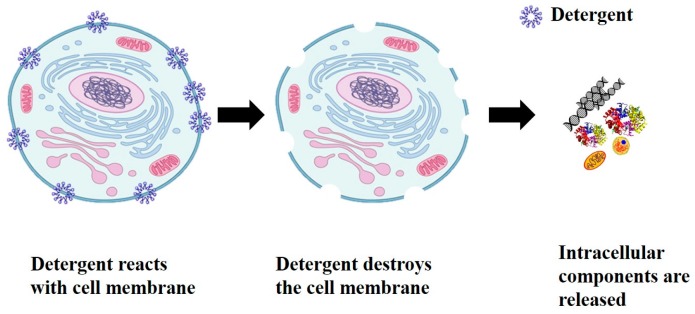
Cell lysis using detergent to open the cell membrane and release the intracellular components. Reproduced with permission from Genomics education program.

Cells are the fundamental unit of all living organisms. Similar to the human body, cells also have a set of organs known as organelles, which are responsible for the cell’s ability to perform various kinds of functions. Additionally, the genetic information for the development and functioning of any organism is encoded in DNA or RNA sequences that are located inside the cell. The cell has an outer boundary called cell membrane, which encloses all the contents. The cell membrane serves as a barrier and regulates the transport of material between the inside and outside of the cell. The cell membrane must be disrupted or destroyed in order to access the DNA from inside the cell for molecular diagnosis, such as to identify pathogens [9]. A schematic representation of the cell lysis procedure is shown in Figure 1 where a detergent is used to disrupt the membrane chemically. Detergents react with cell membrane forming pores on the surface of membrane resulting in release of intracellular components such as DNA, RNA, proteins, etc.

### 2.1. Classification of Cell Types

Cells are of two types: eukaryotic (such as mammalian cells) and prokaryotic (such as bacteria). The main difference between these two types is in their structure and organization.

**Figure 2:**
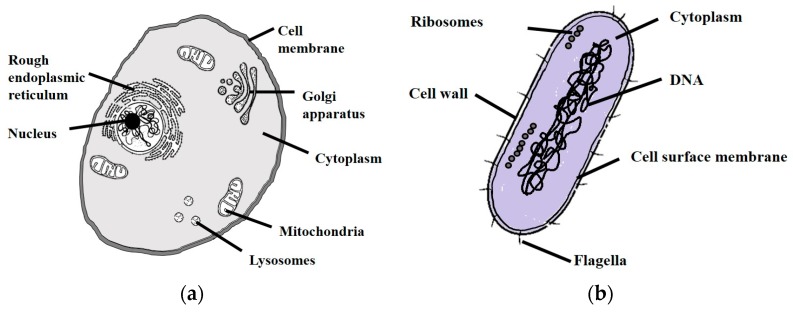
Anatomy of: (**a**) mammalian cell; and (**b**) bacteria.

Figure 2 illustrates the difference between mammalian cells and bacteria. Mammalian cells have a boundary called cytoplasmic membrane that encloses the contents of the cell. In the case of bacteria, there are multiple layers enclosing the cell content and the innermost and outermost of them are called the plasma membrane and cell wall, respectively. Depending on the type of bacteria, the number of these layers varies. In the case of gram-positive bacteria, the plasma membrane is surrounded by another membrane known as cell wall or the peptidoglycan layer, whereas gram-negative bacteria, such as *E. coli*, consist of a cytoplasmic membrane, cell wall and an outer membrane. The composition of these cell layers such as structure and properties, have been extensively reviewed [10,11,12].

#### 2.1.1. Cytoplasmic Membrane

**Figure 3:**
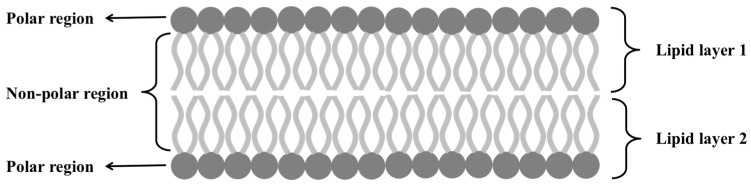
Structure of cell membrane showing the arrangement of hydrophobic (non-polar) and hydrophilic (polar) regions of phospholipid bilayer.

Cytoplasmic membrane also known as plasma membrane is a thin structure which acts as a barrier between internal and external environment of cell. This layer is typically 4-nm thick [13,14]. The plasma membrane is mainly made of a phospholipid bilayer that contains highly hydrophobic (fatty acid) and hydrophilic (glycerol) moieties. Figure 3 shows the hydrophobic and hydrophilic configurations of a cell membrane. When these phospholipids aggregate in an aqueous environment, they try to form a bilayer structure where hydrophobic components point to each other and hydrophilic glycerol remain exposed to the outside environment. Proteins are integrated on the surface of the lipid bilayer. Due to the hydrophobic nature of cytoplasmic membrane, it forms a tight barrier; however, some small hydrophobic molecules can pass through this barrier by diffusion.

**Figure 4:**
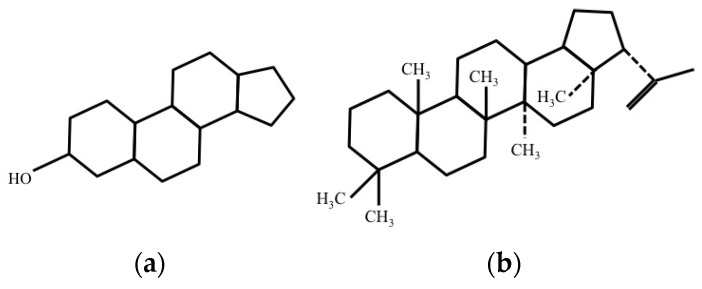
Structure of: (**a**) Sterols; and (**b**) Hopanoids.

Eukaryotic cells have rigid and planar molecules called sterols (Figure 4a) in their membrane. The association of sterols increases the stability of cells and makes them inflexible. On the other hand, sterols are not present in prokaryotic cell. However, hopanoids (Figure 4b), molecules similar to sterols, are present in membrane of various bacterial cells. Similar to sterols, hopanoids increase the stability and rigidity of bacterial membrane.

#### 2.1.2. Cell Wall

Osmotic pressure is developed inside the cell due to the concentration difference of solutes across the membrane. For *E. coli*, this pressure is estimated around 2 atm [15]. To withstand these pressures, bacteria contains a cell wall or peptidoglycan layer, which also contributes to the shape and rigidity of the cell. This layer consists of two sugar derivatives named *N*-acetylglucosamine and *N*-acetylmuramic acid as well as a small group of amino acids consisting of l-alanine, d-alanine and d-glutamic acid.

The basic structure of this peptidoglycan layer is a thin sheet where the aforementioned sugar derivatives are connected to each other by glycosidic bond forming a glycan chain. These chains are cross-linked by amino acid and the whole structure gives the cell rigidity in all directions. The strength of this structure depends on the frequency of chains and their cross linking.

In gram-positive bacteria, peptidoglycan layer makes up 50%–80% of the cell envelope and 10% of this layer is associated with teichoic acid which provides a greater structural resistance to breakage [16]. In contrast, 10%–20% of the cell envelope of gram-negative bacteria is composed of a 1.2 to 2.0 nm thick peptidoglycan layer [16].

#### 2.1.3. Outer Membrane

In addition to the peptidoglycan layer, there is another layer in the gram-negative bacteria known as the outer membrane. This layer is made of lipopolysaccharide which contains polysaccharides, lipids and proteins. It isolates the peptidoglycan layer from the outer environment and increases the structural firmness of the bacteria. The outer membrane is not permeable to enzymes.

While the focus of the paper is the disruption of the cell boundary, this brief discussion regarding types of cells and their bounding structures is critical in selecting the appropriate methods and materials for lysis. In the next section, the different cell lysis techniques are explained.

## 3. Classification of Cell Lysis Methods

**Figure 5:**
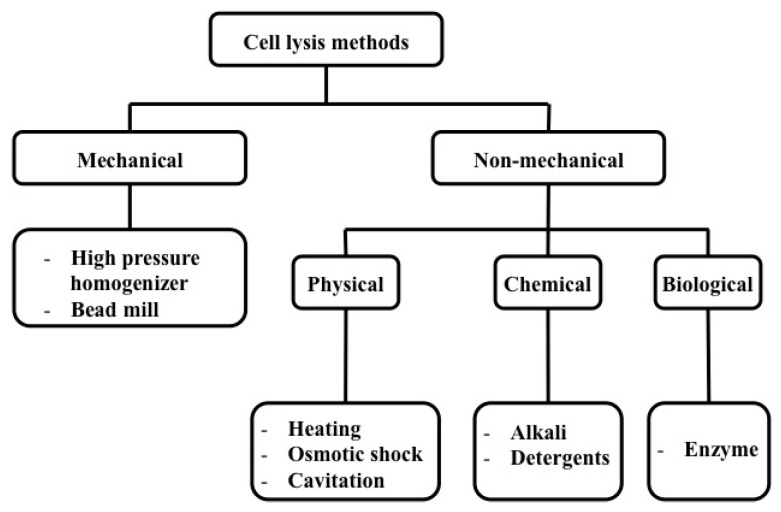
Classification of cell lysis methods.

A number of methods, as depicted in Figure 5, have been established to lyse cells in the macro and micro scale and these methods can be categorized mainly as mechanical and non-mechanical techniques.

### 3.1. Mechanical Lysis

In mechanical lysis, cell membrane is physically broken down by using shear force. This method is the most popular and is available commercially because of a combination of high throughput and higher lysing efficiency. Different types of mechanical lysis techniques are discussed below.

#### 3.1.1. High Pressure Homogenizer

**Figure 6:**
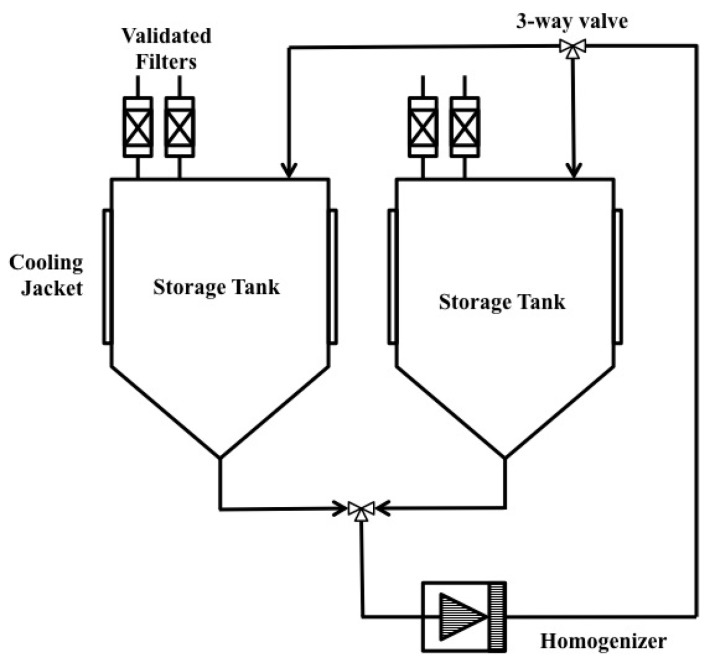
Example of a high pressure homogenizer system. Reproduced with permission from [18].

High Pressure Homogenizer (HPH) is one of the most widely used equipment for large scale microbial disruption. In this method, cells in media are forced through an orifice valve using high pressure. Disruption of the membrane occurs due to high shear force at the orifice when the cell is subjected to compression while entering the orifice and expansion upon discharge. Figure 6 shows an example of a commercially available HPH system. In this system, two storage tanks are employed which alternate the feed and allow for multiple passes of the homogenate. A positive displacement pump is used to draw the cell suspension. Depending on the types of the cells, 15–150 MPa is required [3,17].

Sauer et al. [19] proposed a model to relate the amount of protein released by homogenizer to the applied pressure for *E. coli*.

(1) $$\ln\left(\frac{R_{m}}{R_{m}-R}\right)=KNP^{a}$$

where *R* is protein released, *R*_m_ is the maximum protein available for release, *P* is pressure in MPa, *N* is the number of passes, *K* is the rate constant and *a* is the pressure exponent.

Since the release of protein is independent of biomass concentration, higher concentration of cell can be disrupted at the same time. However, generation of heat is a problem in this method. Cooling systems can be used to minimize the heat generated. Augenstein et al. [20] reported the degradation of some enzymes during homogenization due to the high pressure. A combination of lysis methods, for example chemical treatment along with homogenization, has shown better results [18].

#### 3.1.2. Bead Mill

Bead mill, also known as bead beating method, is a widely used laboratory scale mechanical cell lysis method. The cells are disrupted by agitating tiny beads made of glass, steel or ceramic which are mixed along with the cell suspension at high speeds. The beads collide with the cells breaking open the cell membrane and releasing the intracellular components by shear force. This process is influenced by many parameters such as bead diameter and density, cell concentration and speed of agitator. Smaller beads with a range of 0.25–0.5 mm are more effective and recommended for lysis [3,21]. Using this technique, several kinds of cells can be lysed for example yeast and bacteria [22,23]. Cell membrane can become totally disintegrated by this method confirming that the intracellular molecules are released. Thus, the efficiency of this method of lysing cells is very high. However, complete disintegration produces small cell debris and thereby separation and purification of sample becomes harder. In addition, heat generation occurs in this process due to the collision between beads and cells. This elevated heat may degrade proteins and RNA.

Ho et al. [24] have compared different cell lysis methods for extracting recombinant hepatitis B core antigen from *E. coli*. They concluded that continuous recycling bead milling method is the most effective method in terms of cost and time. They also report that the most effective method for cell disruption was HPH. Table 1 lists the various commercially available mechanical cell lysis instruments on the market.

Goldberg [25] reviewed the different mechanical cell lysis methods available at both laboratory and industrial scale. Some other mechanical techniques such as rotor/stator shear homogenizer, solid pressure shear, impingement jet and colloid mills are also very efficient in rupturing various kinds of cells [3]. In conclusion, mechanical method is a very efficient method to lyse a wide range of cells. However, problems such as heating of sample volume, degradation of cellular products, cell debris and higher cost limit the use of this method.

### 3.2. Non-Mechanical Lysis

Non-mechanical lysis can be categorized into three main groups, namely physical, chemical and biological, where each group is further classified based on the specific techniques and methods used for lysis. A detailed description of each type is presented below.

#### 3.2.1. Physical Disruption

Physical disruption is a non-contact method which utilize external force to rupture the cell membrane. The different forces include heat, pressure and sound energy. They can be classified as thermal lysis, cavitation and osmotic shock.

##### Thermal Lysis

Cell lysis can be conducted by repeated freezing and thawing cycles. This causes formation of ice on the cell membrane which helps in breaking down the cell membrane. This method is time consuming and cannot be used for extracting cellular components sensitive to temperature. Johnson et al. [26] have shown that by using the freeze/thaw cycles they were able to separate highly expressed recombinant proteins from *E. coli.* They submerged the sample solution in dry ice/ethanol bath for 2 min and then thawed in ice/water bath for 8 min. This cycle was repeated three times in total. They compared different cell lysis methods (French press, sonication and enzymatic lysis) and found the freezing/thawing method to be most efficient for extracting these highly expressed proteins. Elevated temperature has also been shown to be capable of cell lysis. High temperature damages the membrane by denaturizing the membrane proteins and results in the release of intracellular organelles. A significant amount of protein can be released from *E. coli* over the temperature range of 90 °C [2,27]. However, heating for a long period may damage the DNA. This method is expensive [28] and so it is not widely used for macroscale industrial applications. In addition, damage of target materials such as protein and enzymes due to higher temperature restricts the use of thermal lysis method. Zhu et al. [29] have described a procedure by modifying the thermal lysis method to extract plasmid DNA from *E. coli* in large quantities (100 mg) in about 2 h. In their method, the *E. coli* are pretreated with lysozyme prior to passing through a heat exchange coil set at 70 °C to lyse the cells. They used peristaltic pump and two heating coils at constant temperature and avoided the use of centrifugation step which enabled them to develop a continuous and controllable flow through protocol for lysing the cells at high throughput and obtaining large quantities of plasmid DNA. Thermal lysis is an attractive method at the micro scale used in many microfluidic devices. The high surface to volume ratio in microfluidic devices helps in cell lysis by quickly dissipating the heat and rupturing the cell membranes effectively. These techniques are covered later in Section 5.

##### Cavitation

Cavitation is a technique which is used for the formation and subsequent rupture of cavities or bubbles. These cavities can be formed by reducing the local pressure which can be done by increasing the velocity, ultrasonic vibration, etc. Subsequently, reduction of pressure causes the collapse of the cavity or bubble. This pressure fluctuation is of the order of 1000 MPa [3].

During the collapse of a bubble, a large amount of mechanical energy is released in the form of a shockwave that propagates through the media. Since this shock wave has high energy, it has been used to disintegrate the cell membrane. Ultrasonic and hydrodynamic methods have been used for generating cavitation used to disrupt cells.

Ultrasonic Cavitation is a widely known laboratory based technique for disruption of the cells. Ultrasonic vibration (15–20 kHz) can be used to generate a sonic pressure wave [5]. It has been shown that disruption is independent of biomass concentration and proportional to power input. This technique also produces very small cell debris which might be a problem for subsequent processes. In addition, large amount of heat is generated which needs to be dissipated. Enzymes that come out from cell after Ultrasonic Cavitation have also been reported to be degraded [30].

To overcome the problems associated with ultrasonic cavitation, such as high power requirement and high energy to dissipate heat problem, hydrodynamic cavitation has been used to disrupt the cell membrane [31]. Hydrodynamic cavitation is produced by pumping the cell suspension through a constricted channel which results in an increase in velocity. Lee et al. [32] have demonstrated the use of hydrodynamic cavitation as an efficient method to disrupt the cell membrane of cells to extract the lipids. They report that the energy required for lipid extraction from cells using the hydrodynamic cavitation technique was 3 MJ/kg which is 10 times more efficient compared to sonication in terms of energy consumption. In another study by Capocellia et al. [33] the acoustic and hydrodynamic cavitation methods were compared for microbial cell disruption. Their simulation results show that the hydrodynamic cavitation is an order of magnitude more efficient than acoustic cavitation method for cell disruption.

##### Osmotic Shock

When the concentration of salt surrounding a cell is suddenly changed such that there is a concentration difference between the inside and outside of the cell, the cell membrane becomes permeable to water due to osmosis. If the concentration of salt is lower in the surrounding solution, water enters the cell and the cell swells up and subsequently bursts. This technique is suitable for mammalian cell due to the fragile structure of membrane; however, periplasmic proteins may be released in the case of gram-negative bacteria [34]. Chen et al. [35] compared osmotic shock method and sonication for recovery of recombinant creatinase from *E. coli.* They found osmotic shock method resulted in a 60% creatinase recovery and 3.9 fold purification compared to sonication. They also observed that when the cells were pretreated with divalent cation (Ca^2+^ or Mg^2+^) the efficiency of osmotic shock method could be improved to 75% and 4.5 fold purification. Another study by Byreddy et al. [36] showed that osmotic shock method resulted in the highest yield of lipids from Thraustochytrid strains when compared to grinding with liquid nitrogen, bead vortexing and sonication methods.

#### 3.2.2. Chemical Cell Disruption

Chemical lysis methods use lysis buffers to disrupt the cell membrane. Lysis buffers break the cell membrane by changing the pH. Detergents can also be added to cell lysis buffers to solubilize the membrane proteins and to rupture the cell membrane to release its contents. Chemical lysis can be classified as alkaline lysis and detergent lysis.

##### Alkaline Lysis

In alkaline lysis, OH^−^ ions are the main component used for lysing cell membrane [37]. The lysis buffer consists of sodium hydroxide and sodium dodecyl sulphate (SDS). The OH^−^ ion reacts with the cell membrane and breaks the fatty acid-glycerol ester bonds and subsequently makes the cell membrane permeable and the SDS solubilizes the proteins and the membrane. The pH range of 11.5–12.5 is preferable for cell lysis [3,38]. Although this method is suitable for all kinds of cells, this process is very slow and takes about 6 to 12 h. This method is mostly used for isolating plasmid DNA from bacteria [39,40].

##### Detergent Lysis

Detergents also called surfactants have an ability to disrupt the hydrophobic-hydrophilic interactions. Since the cell membrane is a bi-lipid layer made of both hydrophobic and hydrophilic molecules, detergents can be used to disintegrate them. Detergents are capable of disrupting the lipid–lipid, lipid–protein and protein-protein interactions. Based on their charge carrying capacity, they can be divided into cationic, anionic and non-ionic detergents. Detergents are most widely used for lysing mammalian cells. For lysing bacterial cells, first the cell wall has to be broken down in order to access the cell membrane. Detergents are often used along with lysozymes for lysing bacteria (e.g., yeast). Table 2 lists all the detergents according to their charge and properties. Out of the three types of detergents, non-ionic detergents are mostly preferred as they cause the least amount of damage to proteins and enzymes. 3-[(3-cholamidopropyl)dimethylammonio]-1-propanesulfonate (CHAPS) and 3-[(3-cholamidopropyl)dimethylammonio]-2-hydroxy-1-propanesulfonate (CHAPSO), a zwitterionic detergent, is one of the most popular non-ionic detergents. Other non-ionic detergents include Triton-X and Tween series. Ionic detergent such as SDS is widely used for lysing cells because of its high affinity to bind to proteins and denature them quickly. It is used in gel electrophoresis and western blotting techniques. The hydrophilic part of an anionic detergent is mostly a sulphate or carboxylic group whereas for cationic detergent it is ammonium group. Apart from ionic and non-ionic detergents, chaotropic agents can also be used for cell lysis. These include urea, guanidine and Ethylenediaminetetraacetic acid (EDTA) which can break the structure of water and make it less hydrophilic and there by weakening the hydrophobic interactions. An additional purification step has to be in cooperated into the cell lysis protocol when using detergents [41].

#### 3.2.3. Enzymatic Cell Lysis

Enzymatic lysis is a biological cell lysis method in which enzymes such as lysozyme, lysostaphin, zymolase, cellulose, protease or glycanase are used. Most of these enzymes are available commercially and can be used for large scale lysis. One advantage of enzymatic lysis is its specificity. For example, lysozymes are used for bacterial cell lysis whereas chitinase can be used for yeast cell lysis and pectinases are used for plant cell lysis. Lysozyme reacts with peptidoglycan layer and breaks the glycosidic bond. For that reason, gram-positive bacteria can be directly exposed to lysozyme, however, outer membrane of the gram-negative bacteria needs to be removed before exposing the peptidoglycan layer to the enzyme. Lysozyme treatment is generally conducted at pH 6–7 and at 35 °C [3]. For gram-negative bacteria, lysozyme is used in combination with detergents to break the cell wall and membrane. Another example is proteinase K which is used for isolating genomic DNA. Andrews et al. [43] and Salazar et al. [44] have reviewed about enzymatic lysis of microbial cells.

### 3.3. Combination of Mechanical and Non-Mechanical Methods

From the aforementioned discussion, it can be concluded that chemical methods make the membrane permeable which is good for selective product release from cells such as protein or enzymes, however complete cell disruption may not be achieved which may be required for release of other products such as nucleic acid or cell debris. In order to overcome this problem, combinations of non-mechanical and mechanical methods have been employed to increase the efficiency of lysis [3,31]. Anand et al. [45] have studied the effect of combination of mechanical and chemical cell lysis methods on the extent of recovery of intracellular products. They used EDTA as a pretreatment method combined with high pressure homogenizer. The pretreatment lowered the pressure from 34.5 to 13.8 MPa in high pressure homogenizer for recovery of proteins from *E. coli*. cells. They also conclude that pretreatment with guanidium hydrochloride and Triton X-100 resulted in an increase in intracellular release with decrease in usage of energy.

### 3.4. Overview and Comparison of Different Cell Lysis Methods

A comparison between different types of cell lysis techniques (mechanical and non-mechanical) is summarized in Table 3. It also provides an overview of the major commercial as well as laboratory based lysis techniques with advantages and disadvantages associated with each method.

## 4. Microfabricated Platforms for Cell Lysis

Microfluidics is one of the emerging platforms for cell lysis on a micro scale. Microfluidics is the manipulation and handling of small volumes (nano- to picoliters) of liquid in microchannels. Due to the micro scale operation regime, microfluidics is well suited for application where the sample or sample volume is small. This lowers the cost of the analysis due to low consumption of reagents [46]. Microfluidics also enables integration of different modules (or operations) into one device. For example, cells can be lysed and the intracellular products can directly be post processed (PCR or DNA isolation for diagnostics) inside the same device [47,48]. Although there have been a number of reviews on cell lysis in the past 10 years [7,8,49], some of the recent developments in the field have not been reviewed. This review will focus on the recent developments from 2014 onwards and will briefly cover the developments from before, which have been extensively surveyed. Some of the macro scale techniques have been implemented in microfabricated devices for cell lysis. Techniques such as electrical lysis methods are applicable only in the micro scale. Microfluidic lysis technology can be broadly classified into six types. They include mechanical lysis, thermal lysis, chemical lysis, optical lysis, acoustic lysis and electrical lysis.

### 4.1. Mechanical Lysis

**Figure 7:**
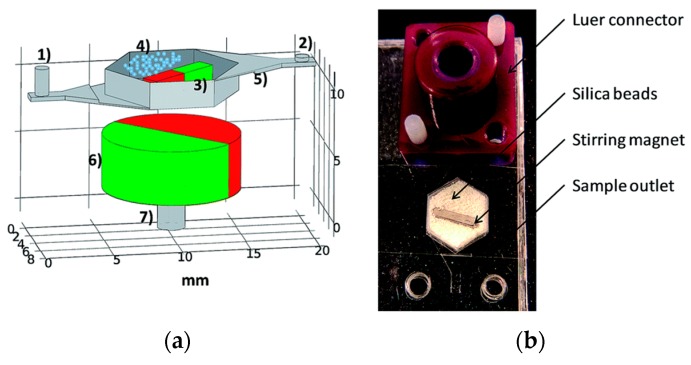
Miniaturized bead beading cell lysis system: (**a**) various components: (1) inlet; (2) outlet; (3) stirring magnet; (4) zirconia/silica beads; (5) bead weir; (6) rotating magnet; and (7) electric motor coupling; and (**b**) image of the device for lysis. Reproduced with permission from [50].

Mechanical lysis in microfluidics involves physically disrupting the cell membrane using shear or frictional forces and compressive stresses. Berasaluce et al. [50] developed a miniaturized bead beating based method to lyse large cell volumes. Zirconium/silica beads were placed inside a cell lysis chamber along with a permanent magnet and actuation of an external magnetic field caused the motion of the beads inside the chamber. Figure 7 shows the various components and device assembled for cell lysis. *Staphylococcus epidermidis* cells were used in this study and they studied the effect of bead size, volume, flow rate and surfactant (Tween-20) on lysing efficiency. They found the optimum parameters achieved a 43% higher yield efficiency at a flow rate of 60 μL/min compared to off chip bead beating system.

**Figure 8:**
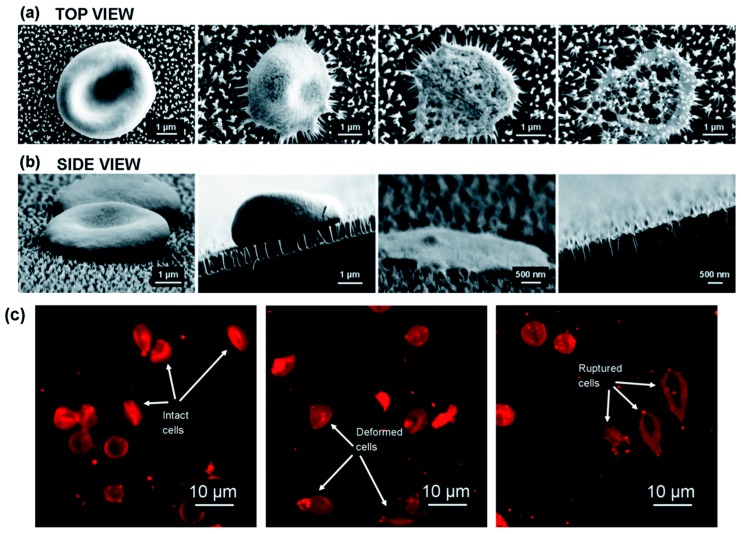
Cell lysis using nano pillars: (**a**,**b**) top and side view of the cells interacting with the nanopillars; and (**c**) confocal laser scanning microscopy pictures of intact, deformed and ruptured cells. Reproduced with permission from [51].

Pham et al. [51] have recently used nanotechnology to fabricate black silicon nano pillars to lyse erythrocytes in about 3 min. They fabricated these nanopillar with ~12 nm tip diameter and 600 nm tall on silicon substrate using reactive ion etching technology. The authors showed that the interaction of erythrocytes cultured on nanopillar arrays causes stress induced cell deformation, rupture and lysis in about 3 min. Figure 8 shows the interaction of erythrocytes with the nanostructures.

**Figure 9:**
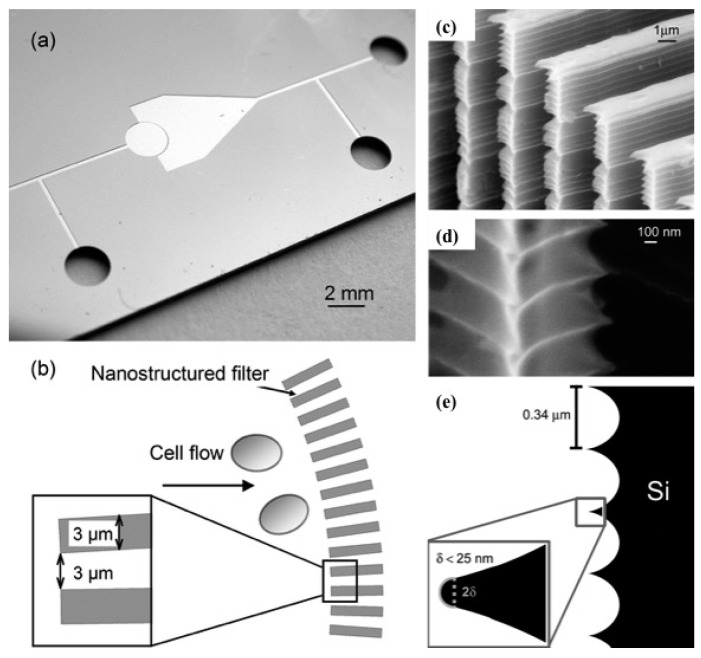
Mechanical lysis using nanoscale barbs: (**a**) microfluidic device showing different inlets and outlet channels; (**b**) schematic of the barbs; (**c**) deep reactive ion etching (DRIE) fabricated nano-knives; (**d**) magnified image of nano-knives patterned using DRIE technique and (**e**) dimensions of the nano-knives used for cell lysis. Reproduced with permission from [52].

Mechanical lysis has been demonstrated by using nano-scale barb [52]. When cells are forced through small opening, high shear forces cause rupture of the cell membrane. Similar principle has been used here where “nanoknives” were fabricated in the wall of microchannels by using modified deep reactive ion etching (DRIE). Distance between these sharp edges was 0.35 μm and width of the channel was 3 μm. The lysis section of this device consisted of an array of these “nanoknives” patterned on a microchannel as shown in Figure 9b. Human promyelocytic leukemia cells (HL-60) were used to pass through this section at sufficient velocity. The addition of this “nanoknives” pattern increased the amount of lysis. This device was used to extract protein from inside the cell. It has been estimated that as much as 99% of the cell was lysed but, only 6% protein was released.

Alternatively, mechanical impingement through collision has also been used to lyse in the microscale [53,54,55]. Cells were suspended in solution with glass beads and placed on the microfluidic compact disc (CD) device, which was then set to rotate at a very high velocity. The centrifugal force generated by the rotation, causes collision and friction between cells and beads, which results in cell lysis. Various kinds of cells including mammalian, bacteria and yeast have been lysed using this technique.

Though the efficiency of the mechanical lysis is very high, these disruption methods have some drawbacks in microscale application. Fabrication of these devices is complex as well as expensive and collecting the target materials from a complex mixture is very difficult.

### 4.2. Thermal Lysis

In thermal lysis, heat is supplied to the cells to denature the membrane proteins and lyse the cells. One advantage of thermal lysis is the easy integration of microfluidic devices such as polymerase chain reaction (PCR). The thermal lysis can be performed in such devices with no additional modification. The cells are generally heated above 90 °C and the intracellular products are cycled through different temperatures for example in a PCR device. Tsougeni et al. [56] fabricated a microfluidic device which can capture and lyse cells. They used thermal lysis at 95 °C for 10 min to capture and lyse bacteria. Nanostructures were fabricated in poly(methyl methacrylate) using lithography and plasma etching technique. Microfluidic PCR devices which have incorporated thermal cell lysis [57,58,59] consist of a glass chamber and a resistive heater to heat the chamber.

In general, thermal lysis is effective in a microfluidic platform, however, these devices are not suitable for sample preparation where the sample is of a large volume and cells have to be lysed from a continuous flow [29]. However, cells have to be treated with lysozyme in order to break the cell wall and make bacteria protoplast. The addition of this lysozyme is time consuming and requires complex structures. Moreover, preserving the enzyme within the device becomes problematic when the device has to be used for a long period of time. Higher lysis time and elevated power consumption are other drawbacks of this method.

### 4.3. Chemical Lysis

Chemical lysis methods use chemical reagents such as surfactants, lysis buffers and enzymes to solubilize lipids and proteins in the cell membrane to create pores and lyse cells. Although chemical and enzymatic methods are categorized separately in macro scale method, these two techniques are incorporated in the same group for micro scale cell lysis techniques. Buser et al. [60] lysed gram-positive bacteria (Staphylococcus aureus) and RNA virus (respiratory syncytial virus) using a dried enzyme mixture (achromopeptidase). They were able to lyse in less than a minute and then used a disposable chemical heater to deactivate the lysis enzyme. They were able to amplify (off-chip) the lysate without purification and showed the proof of principle for a point of care device for diagnostics.

Kashyap et al. [61] developed a microfluidic probe for selective local lysis of adherent cells (~300 cells) for nucleic acid analysis. Hall et al. [62] used a device for cell lysis experiment, which had two supply wells and a pressure well. Mixing of cell and lysis solution was controlled by adjusting the pressure of the wells. Three different types of solution were used—Solution A containing only SDS (detergent based reagent), Solution B containing surfactant, Triton X-100, Tween-20 with enzyme such as lysozyme, protease, proteinase K and Solution C containing an antibiotic named polymyxin B. Gram-negative and gram-positive bacteria were used for lysis. It was concluded that detergent alone was not suitable for lysis, while Solution B, a mixture of chemical surfactants and biological reagents, can disintegrate the cell membrane and lyse various kinds of bacteria. However, polymyxin B can be potentially used in microfluidic cell lysis platform only for gram-negative bacteria.

**Figure 10:**
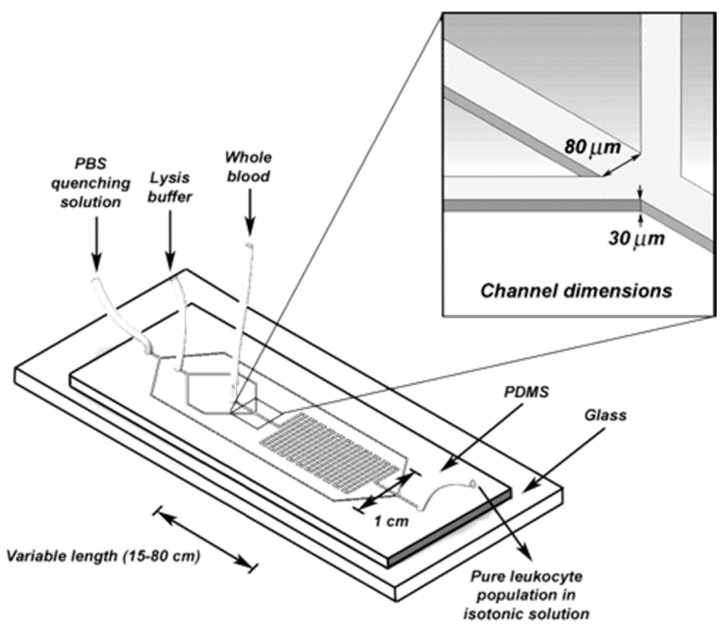
Schematic of a simple chamber and serpentine microfluidic channel for chemical lysis. Reproduced with permission from [65].

Kim et al. [63] also developed a microfluidic device with two inlets and outlets in order to develop an optimal lysis reagent for gram-negative bacteria. Heo et al. [64] demonstrated a microfluidic based bioreactor which was capable of entrapping *E. coli* by using hydrogel patches. Then the immobilized *E. coli* was lysed by using SDS as it can penetrate hydrogel. Cell lysis was accomplished within 20 min. This device was capable of cell lysis using only SDS, however, the previous one could not due to lower exposure time in chemical environment. In another study, Sethu et al. [65] also developed a microfluidic chip (Figure 10) to lyse Erythrocyte in order to isolate Leukocyte. One hundred-percent recovery was possible within 40 s. The device consists of three inlet reservoirs and one outlet reservoir. One inlet was used to flow the entire blood. Second inlet was used for lysis buffer containing mainly aluminum oxide and two side channels were connected with this inlet which converged to direct the entire blood into a narrow stream. This increases the surface contact between the lysis buffer and the cells. The mixture of cells and lysis buffer was then run through a long channel with a number of “U” turns to enhance the buffer. Finally, third inlet was used to flow the phosphate buffer in order to dilute the sample for restoring the physiological concentration [66,67].

Even though chemical lysis method is widely used in many microfluidic devices, this method requires an additional time consuming step for reagents delivery. Therefore, complex microfluidics structures including injection channels and micro-mixers to homogenize the samples are needed [66,68]. After lysis, these reagents might interfere with downstream assay as it is very hard to separate the target molecules [69]. In addition, storage of these reagents is a problem which is why the device cannot be used for long time.

### 4.4. Optical Lysis

Optical lysis of cells involves the use of lasers and optically induced dielectrophoresis (ODEP) techniques to break open the cell membrane. In laser lysis, a shock wave created by a cavitation bubble, lysis the cell membrane. A focused laser pulse at the cell solution interface creates this cavitation bubble. In ODEP, a conductive electrode and a photoconductive layer (for example amorphous silicon) are formed on the top surface of glass slide. A non-uniform electric field is generated by shining light on the photoconductive layer which then generates a transmembrane potential across the cell membrane disrupting the cell membrane. Huang et al. [70] developed an optically induced cell lysis microfluidic chip for lysing HEK293T cells and extracting intact nucleus. They report cell lysis and nucleus separation efficiency as 78% and 80% respectively using this device.

**Figure 11:**
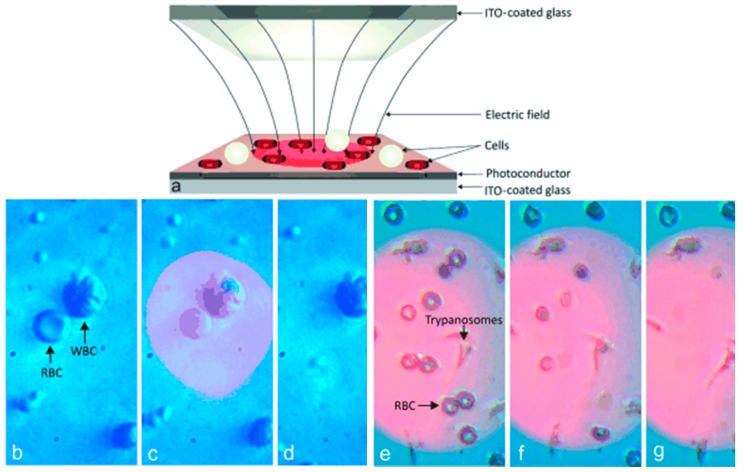
Optical cell lysis device: (**a**) cell lysis chip using optically induced dielectrophoresis (ODEP); (**b**–**d**) cell lysis of red blood cells in a mixture of white and red blood cells; and (**e**–**g**) lysis of red blood cells in a mixture of red blood cells and trypanosomes. Reproduced with permission from [71].

Kremer et al. [71] lysed cells using an opto-electrical setup. They were able to lyse cells selected based on shape of the cell. They used ODEP to lyse red blood cells in a mixture of red and white blood cells. They developed a method that enabled shape-selectivity such that cells with a different geometry will lyse in a mixture of cell types. The cell with a different shape induces a non-uniform electric field which is used for lysis. Figure 11 shows the schematic of the lysis chip and lysis of differently shaped cells.

Use of laser light to induce lysis has also been attempted in microfluidic devices. In one instance, optical lysis was induced by application of a nanosecond 532 nm laser pulse [72] which generates a microplasma locally. The plasma collapses causing cavitation, bubble expansion and its collapse as described in previous section are the main reason for a laser induced cell lysis. Various types of cell lines such as rat basophilic leukemia (RBL) [73], rat-kangaroo (Potorous tridactylis) epithelial kidney cells (PtK2) [74], and murine interleukin-3 dependent pro-B (BAF-3) [75] have been lysed by using this laser induced method. However, all these experiments had been done for single cell analysis. It has been found that when laser based lysis was incorporated with polydimethylsiloxane (PDMS) microchannel efficiency of lysis decreased [75]. It was suggested that this may be due to the deformation of PDMS walls which dissipates the mechanical energy from the bubble collapse. For that reason, high energy was required.

Ultraviolet (UV) light array combined with titanium oxide has been used to lyse the cell [76]. Titanium oxide possesses photolytic properties and excitation energy that falls within UV range. When titanium oxides are excited with UV light array, electrons in the valence band are excited to conduct ion band which results in electron–hole pairs. In aqueous environment, these electron–hole pairs react with surrounding molecules and generate free radicals such as OH, O and O_2_^−^. These react with cell membrane and lyse the cell. *E. coli* cells were lysed with the above technique. A primary disadvantage of ultraviolet lysis was that the time required to lyse the cell was very high (45 min).

### 4.5. Acoustic Lysis

**Figure 12:**
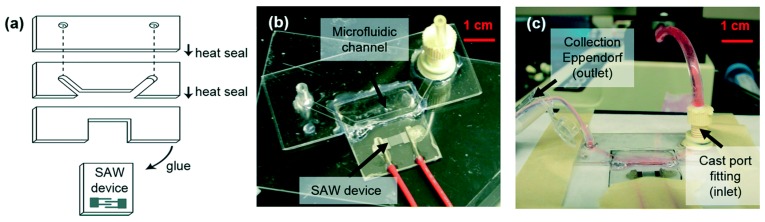
Surface acoustic wave (SAW) lysis microfluidic device: (**a**) assembly of device; and (**b**,**c**) as fabricated device with liquid inlet and outlet for exosome lysis. Reproduced with permission from [77].

In acoustic lysis, a high energy sound wave is generated which is used for cell lysis. This surface acoustic wave (SAW) is produced on a piezoelectric substrate. An inter-digitated transducer (IDT) can be used to produce a SAW electrically with the wave propagating on the surface away from it. Taller et al. [77] have used on chip surface acoustic wave lysis for detecting exosomal RNA for pancreatic cancer study. They achieved a lysis rate of 38% using this technique. Figure 12 shows the fabricated device with the SAW transducer.

They report that the lysis of exosomes is possible due to the effects of acoustic radiation force and dielectric force acting on small particles [78,79]. The SAW device was fabricated using standard photolithography technology. Twenty pairs of titanium aluminum electrodes were patterned on top of piezoelectric lithium niobate substrate to form a single phase unidirectional SAW transducer. This transducer can generate SAW in only one direction. Raw media was exposed to SAW for 30 s at 1 W of power for lysing. The authors report that a lysis efficiency of 38% achieved using this method was sufficient for obtaining enough exosome RNA for detection.

Marentis et al. [80] lysed the eukaryotic cell as well as bacteria by using sonication. This device consists of a microfluidic channel with integrated transducer. The channel was made on glass substrate and piezoelectric transducer was made by depositing zinc-oxide and gold on quartz substrate. The transducers were driven by a sinusoidal source in the 360-MHz range. Eighty-percent lysis of HL-60 and 50% lysis of Bacillus Subtilis spores were obtained by using this device. The temperature rise due to sonication was moderated by using ice pack and cold finger. Ultrasonic horn tip and liquid region are coupled in a microfluidic chip by increasing fluidic pressure in order to increase the efficiency of lysis [81].

Reboud et al. [82] have developed a disposable microfluidic chip to detect the rodent malaria parasite Plasmodium berghei in blood. They used SAW to lyse the red blood cells and parasitic cells in a drop of blood. They report a cell lysis efficiency of more than 99.8% using their device. Xueyong et al. [83] have fabricated a SAW microfluidic device which can lyse red blood cells with high efficiency (95%).

However, sonication has limitations such as generation of heat, complex mechanism as well as expensive fabrication process. Due to this excessive heat generation denaturation of protein and excessive diffusion of the cell contents have been observed [8,84]. To reduce the operation time, cells were first treated with some weak detergent such as digitonin [8,85] before ultrasonic exposure. Digitonin weakened the cell membrane and facilitated lysis.

### 4.6. Electrical Lysis

In electrical method, cells are lysed by exposing them to a strong electric field. An electric field is applied across the cell membrane which creates a transmembrane potential. A potential higher than the threshold potential is required to form pores in the cell membrane. If the value of the potential is lower than the threshold potential, the pores can be resealed by the cell. On the other hand, a high enough potential can completely disintegrate the cell. At such high voltages, it is found that the electric field does not have any effect on the intracellular components [86]. Electric field is the critical parameter to lyse the cell. As higher electric field is required for cell lysis, high voltage generator is required in order to generate this high electric field in macroscale. Thus, this method is not common in macroscale. However, in microscale due to small size of the devices, higher electric field can be obtained at lower voltage. For this reason and as a method for fast and reagentless procedure of lysis, electrical lysis has achieved substantial popularity in microfluidic community.

**Figure 13:**
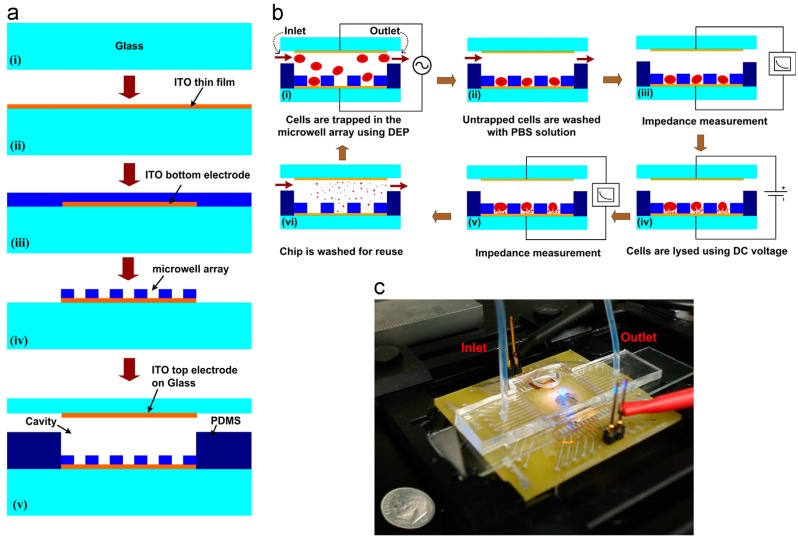
Electrical cell lysis device: (**a**) fabrication protocol of the device; (**b**) working principle of the device; and (**c**) microfluidic device used in the study for lysing red blood cells. Reproduced with permission from [87].

Ameri et al. [87] used a direct current (DC) source to lyse cells in a microfluidic chip. Figure 13 shows the fabrication and working principle of their chip. Their device consists of a glass slide coated with indium tin oxide coating patterned for electrodes. The 6400-Microwell arrays are fabricated using SU-8 polymer by photolithography technique. Inlet and outlet channels are created using PDMS polymer and is sealed using a glass slide with ITO electrode for impedance measurement. Red blood cells (10^7^ cells/mL) are flown through the device at 20 μL/min and dielectrophoresis (DEP) is used to immobilize the cells into the microarray. A DC voltage of 2 V for 10 s was applied to the cell for lysis. The lysis process was monitored using impedance measurement before and after lysis and a decrease in impedance suggested a complete lysis of cells. They report a lysis efficiency of 87% in their device. The authors proposed a device for cell lysis by electric fields and optical free monitoring of the lysis process on a microfluidic platform which could have potential use in the medical diagnostic field.

**Figure 14:**
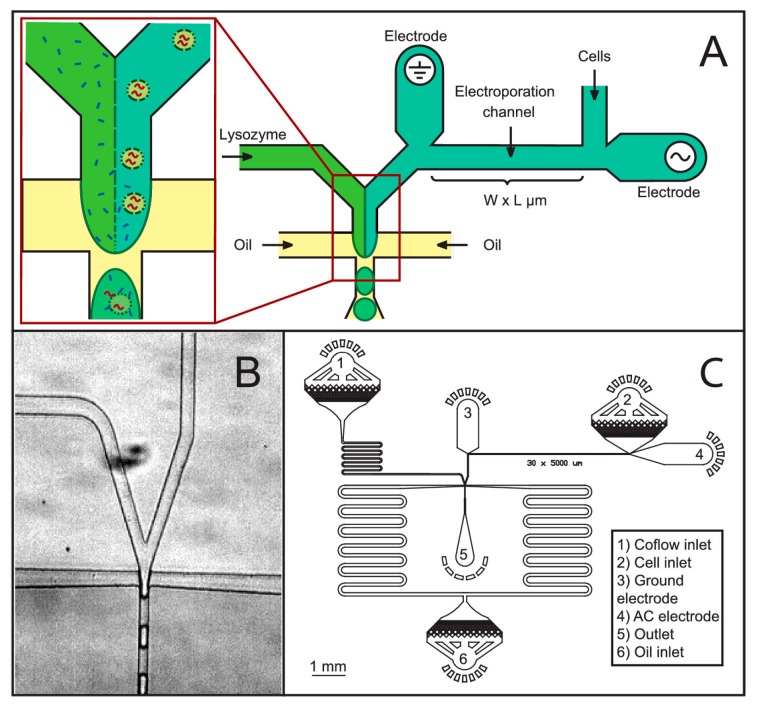
Electrical cell lysis microfluidic device: (**A**) schematic of the electrical lysis and coflow droplet generation microfluidic chip; (**B**) actual image of the droplet generation part; and (**C**) complete electrical lysis with electroporation channels. Reproduced with permission from [89].

Jiang et al. [88] developed a low cost microfluidic device for cell lysis using electric fields. They applied a 10 V square pulse to lyse cells at 50% efficiency. They report a device which had the capability to lyse cells at a much lower voltage compared to a commercially available electropolator device which operated at 1000 V to lyse 200 μL of PK15 cells. They observed bubble formation in their device during cell lysis due to joule heating effect. De Lange et al. [89] have lysed cells in droplets using electric fields. They demonstrated a robust new technique for detergent free cell lysis in droplets. In their device, electric field was applied to lyse bacteria immediately before merging the cell stream with lysozyme and encapsulating the mixture in droplets. They report that with lysozyme alone the lysis efficiency is poor (less than 50%) but when combined with electric fields they were able to obtain up to 90% cell lysis efficiency. Figure 14 shows their microfluidic device for cell lysis in droplets. The authors suggest that their device could be used in applications where use of cell lysis detergents could hinder the cell analysis such as binding assays or studying the chemical activity of proteins and in mass spectroscopy studies where chemical lysis agents can hamper the results.

**Figure 15:**
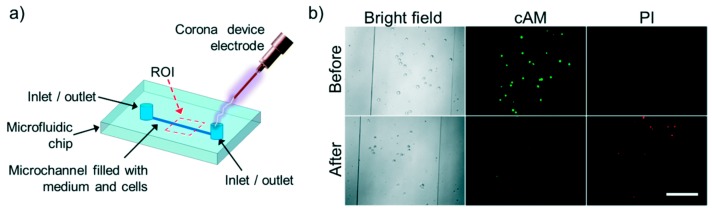
Electrical lysis through handheld plasma device: (**a**) schematic of the device. Cells were lysed using a hand held corona device by applying electric field at the inlet of the device; (**b**) bright field and fluorescent images of before and after of lysis of K562 cells. Reproduced with permission from [90].

Escobedo et al. [90] showed electrical lysis of cells inside a microfluidic chip using a hand held corona device. They were able to lyse baby hamster kidney cells (BHK), enhanced green fluorescent protein human-CP cells (eGFP HCP) 116 and non-adherent K562 leukemia cells completely inside a microfluidic channel. A metal electrode was embedded inside the channel which was used to discharge 10 to 30 kV to lyse the cells in less than 300 ms. Lysis was assessed by observing before and after images of cells using bright field and high speed microscope and also by cell-viability fluorescence probes. They also report no bubble formation during lysis indicating no joule heating effect thereby making this method suitable for analyzing sensitive proteins and intracellular components. Figure 15 shows the setup and results of the study.

Besant et al. [91] detected mRNA molecules of *E. coli* by electrochemical lysis technique. They applied a potential of 20 V, which initiated the cell lysis by producing hydroxide ions from water at cathode to break down bacterial membranes. The sensor electrodes were placed 50 μm away which was enough to detect the mRNA molecules in 10 min. They reported lysis and detection of *E. coli* mRNA at concentrations as low as 0.4 CFU/μL in 2 min which was relevant for clinical application in both sensitivity and time.

**Figure 16:**
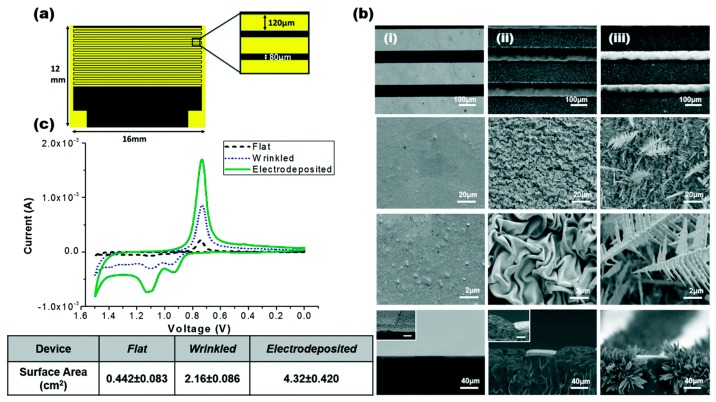
Bacterial lysis device: (**a**) schematic of the lysis device; (**b**) scanning electron micrographs of: (**i**) planar; (**ii**) wrinkled; and (**iii**) electrodeposited electrodes; (**c**) cyclic voltammetry scan of the electrodes. Reproduced with permission from [92].

Gabardo et al. [92] developed a low cost and easy method to fabricate multi-scale 3D electrodes that could be used for bacterial lysis using a combination of electrical and electrochemical means. These micron-sized electrodes can be rapidly prototyped using craft cutting, polymer induced wrinkling and electro-deposition techniques. They report that these tunable electrodes performed better as compared to lithographically prepared electrodes. They were able to successfully extract nucleic acids extracted from lysed bacteria on a microfluidic platform. They reported 95% lysis efficiency at 4 V using their electrodes. Figure 16 shows the device and electrode structures.

Li et al. [93] developed a double nano-electrode electrical cell lysis device to lyse single neuronal cells. Similarly, Wassermann et al. [94] showed cell specific lysis of up to 75% of the total human blood cells using SiO_2_ passivated electrical cell lysis electrodes at an applied voltage of 8–20 V. Ma et al. [95] reported a 10–20-fold increase in mRNA extracted from *M. smegmatis* using electrical lysis in a microfluidic platform as compared to a commercial bead beading instrument. They used a 4000–8000 V/cm field intensity to lyse the bacteria with long pulses (5 s). They report that their device can be effective for mRNA release from hard to lyse cells.

**Figure 17:**
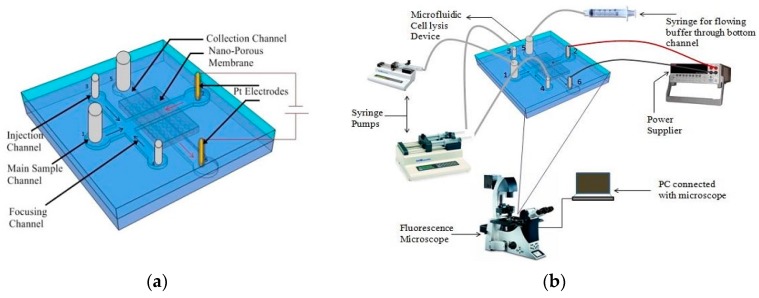
Electrical cell lysis microfluidic device: (**a**) schematic of cell lysis device; and (**b**) experimental setup. Reproduced with permission from [96].

Islam et al. [96] showed the proof of concept of a simple microfluidic device for electrical lysis of larger volumes of sample. They used a nanoporous membrane sandwiched between two microfluidic channels to trap and lyse *E. coli *bacteria by applying 300 V. They report a lysis efficiency of 90% in less than 3 min. Figure 17 shows the schematic of the device used for lysis in their study.

Different types of voltages such as alternating current (AC) [97,98], DC pulses [99,100,101] and continuous DC voltages [102] have been used in order to lyse the cells. Along with electric field, exposure time of cells within that electric field is also an important parameter for cell lysis. It has been found that cells can be lysed by using higher electric field for short period of time as well as lower electric field for long period of time [103]. For that reason, AC and DC pulses of a higher electric field are needed as compared to a continuous DC electric field. As the electric field depends on the distance between the electrodes, microfabricated electrodes have been used during AC or DC pulses. An overview of different electrical lysis devices and the characteristics of the designed system is presented in Table 4.

Lu et al. [104] developed a microfluidic electroporation platform in order to lyse human HT-29 cell. Microfabricated saw-tooth electrode array was used in order to intensify the electric field periodically along the channel. Seventy-four-percent efficiency was obtained for an operational voltage of 8.5 V. However, this mode of lysis is not suitable for bacteria due their sizes and shapes. Compared to mammalian cell, high electric field and longer exposure is needed to lyse bacteria. Rosa [105] developed a chip to lyse bacteria consisting of an array of circular gold electrodes. DC pulses were used and lysis with 17% efficiency was achieved by using an operational voltage 300 V. This efficiency was increased up to 80% after adding enzyme with cell solution. In 2006, Wang et al. [107] proposed application of continuous DC voltage along the channel for cell lysis. The device consists of a single channel with uniform depth and variable width. Since the electric field is inversely proportional to width of the channel, high electric field can be obtained at the narrow section of the channel. Thus, lysis occurs into a predetermined portion of the device. Exposure time of the cell to the electric field can be tuned by changing the length of this narrow section. The configuration of the device was optimized and lysis of complete *E. coli* bacteria was possible at 930 V. Complete disintegration of cell membrane was observed when the electric field was higher than 1500 V/cm. This device was very simple and did not need any microfabricated electrodes. Pt wires were used as electrodes. Only a power generator was needed to operate it. However, bubble generation and Joule heating issue could not be completely eliminated. Similar kind of device was used by Lee [102] where the length and width of the narrow section was modified in order to lyse mammalian cell. Bao et al. [108] also developed a device to lyse *E. coli* by using DC pulses. Release of intracellular materials was observed when the electric field was higher than 1000 V/cm.

In conclusion, electrical method offers a simple, fast and reagent less lysis procedure to lyse various kinds of cells. This method is also suitable for selective lysis and is compatible with other downstream assays such as amplification and separation. Although requirement of high voltage is a problem in this procedure, it can be overcome by decreasing the gap between electrodes through microfabrication. However, heat generation and formation of bubble is a major problem for electric lysis method.

### 4.7. Comparison of Different Microfluidic Technologies for Cell Lysis

Various microfluidic technologies for cell lysis are compared in Table 5. The advantages and disadvantages of different methods are listed for each technique.

## 5. Single Cell Lysis

Single cell analysis has gained much popularity in the recent years owing to the development of new technology. Single cell analysis can be used to understand the cellular heterogeneity in a cell culture as well as used in popular areas of genomics, transcriptomics, proteomics and metabolomics. Single lysis is one of the first steps involved in single cell analysis of intracellular components (proteins, enzymes, DNA, etc.). Many different platforms have been used to study single cell lysis including microfluidics, high speed imaging, capillary electrophoresis and PCR. Cell lysis methods such as laser pulse, nanoscale barbs, acoustic, electrical and chemical (detergents and enzymes) have been utilized to lyse cells. Brown et al. [8] have reviewed single cell lysis methods extensively.

Single cell lysis buffers offered commercially are optimized for single cell RNA extraction. These buffers are designed to reduce sample loss and are compatible with enzymatic reactions such as reverse transcription. Single cell lysis buffers are commercially available from companies such as Thermo Fisher Scientific Inc. (Runcorn, UK), Takara Bio Company (Otsu, Japan), Bio-Rad Laboratories Inc. (Hercules, CA, USA), Signosis (Santa Clara, CA, USA), etc. Companies such as Fluidigm, Dolomite Bio and Molecular Machines and Industries (MMI) specialize in single cell lysis equipment for genomics studies. Svec et al. [109] compared 17 different direct cell lysis protocols for transcript yield and compatibility using quantitative real time PCR method. They concluded that bovine serum albumin (BSA) resulted in the best lysis reagent which resulted in the maximum lysis efficiency and high RNA stability. Kemmerling et al. [110] designed a microcapillary electrode to lyse single cells using electrical pulses. The cell lysates were aspirated into the microcapillary to be later analyzed directly in a transmission electron microscope for protein analysis. Developments in single cell analysis technologies have opened up new possibilities and discoveries in the area of genomics and proteomics.

## 6. Summary

This review provides an overview of cell lysis techniques in the macro and micro scale. The macroscale cell lysis techniques are well established and commercialized by many companies. These techniques include mechanical, chemical, physical and biological techniques. On the other hand, microscale and single cell lysis techniques have recently evolved and use the same macroscale principles for lysis in a miniaturized device. The choice of cell lysis method depends on the type of cells, concentration, application (post processing) and efficiency required. It is difficult to choose one technology, since each method has its own advantages and disadvantages. This review provides a guideline for researchers to choose the cell lysis technology specific for their application. As novel fabrication techniques are introduced in the microfluidics field, we will see better cell lysis techniques with higher efficiency and faster lysis times at reduced cost.

## Figures and Tables

**Table 1:** List of commercially available mechanical cell lysis instruments.

Technique	Trade Names	Website
**High pressure homogenizer**	DeBEE series	www.beei.com
	Microfluidizer	www.microfluidicscorp.com
	Homogenizers	www.gea.com
	French press G-M	www.glenmills.com
**Bead mill**	Bead ruptor	www.omni-inc.com
	DYNO MILL	www.glenmills.com
	TissueLyser II	www.qiagen.com
	Beadmill	www.beadsmill.com
	Mixer mill	www.retsch.com
	Mini bead beater	www.biospec.com

**Table 2:** List of some detergents and their properties. A comprehensive list of detergents can be found here [42].

Detergent	Charge	Properties
**Sodium dodecyl sulphate (SDS)**	Anionic	Strong lysis agent. Good for most cells. Not suitable for sensitive protein extraction.
**Triton X (100, 114)**	Non-ionic	Mild lysis agent. Good for protein analysis.
**NP-40**	Non-ionic	Mild lysis agent. Good for isolating cytoplasmic proteins but not nuclear proteins.
**Tween (20, 80)**	Non-ionic	Mild lysis agent. Good for cell lysis and protein isolation.
**Cetyltrimethylammonium bromide (CTAB)**	Cationic	Generally used for isolating plant DNA.
**CHAPS, CHAPSO**	Zwitterionic	Mild lysis agent. Good for protein isolation.

**Table 3:** Overview and comparison of cell lysis techniques.

Methods	Equipment and Technique Used	Advantages	Disadvantages
**Mechanical**	- High pressure homogenizer - Bead mills	- High efficiency - Is not cell dependent	- Heat generated could damage intracellular products - Expensive method - Difficult to purify the lysed sample
**Non-mechanical**	**Physical**		
	Thermal lysis	- Independent of the cell type - Easy to implement	- Expensive - Damage to proteins and intracellular components
	Cavitation	- Independent of cell type - Large scale integration possible - Operates at a lower temperature and energy level	- Expensive technology - Can cause damage to sensitive proteins - Difficult to purify sample from debris
	Osmotic shock	- Can be used for extracting sensitive intracellular products	- Not suitable for all cell types
	**Chemical**		
	Alkaline lysis	- Suitable for extraction of sensitive intracellular components (proteins, enzymes, DNA) - Suitable for all kinds of cells	- Slow process (6–12 h)
	Detergent lysis	- Suitable for protein release	- Not suitable for isolating sensitive enzyme and proteins-Expensive reagents - Removal of chemical reagent from sample after lysis is difficult - Lower efficiency as complete lysis is not possible
	**Biological**		
	Enzymatic lysis	- Can be very specific for cell types - Suitable for extracting proteins	- Complete lysis not possible - Expensive reagents - Has to be used in combination of detergents for bacteria.

**Table 4:** Different electrical lysis devices used for cell lysis.

Reference	Species	Type of Cell	Cell Size (μm)	Electrode	Type of Voltage (AC/DC)	Lysing Voltage (V)
**[104]**	Human	HT-29	10	Gold	AC	8.5
**[97]**	Human	A431	10	Gold	AC	20
**[98]**	-	FITC-BSA laden vesicle	50	ITO	AC	5
**[99]**	-	Leukocytes	-	3D	DC pulse	10
**[100]**	Human	Red blood cells	6–8	Pt wire	DC/AC	30–170
**[105]**	Bacteria	*E. coli*	-	Gold	DC pulse	50
**[106]**	Hamster	CHO	10–16	Pt wire	DC pulse	1200
**[106]**	Bacteria	*E. coli*		Pt wire	DC	930
**[102]**	Human	Red blood cells	6–8	Pt wire	DC	50
**[87]**	Human	Red blood cells	6–8	ITO	DC	2
**[96]**	Bacteria	*E. coli*	-	Pt wire	DC	300

**Table 5:** Comparison of different microfluidic lysis methods. Cell lysis efficiency was determined by averaging the lysis efficiencies from the references cited. Low: 0%–50%; Medium: 50%–80%; High: 80%–100%.

Lysis Method	Lysis Time	Efficiency	Pros and Cons
**Mechanical**	30 s–10 min	Medium	- Can lyse any type of cell - Robust and can be used for tough cells - Complex device fabrication - Expensive method
**Thermal**	2–5 min	High	- Easy to integrate into device - Low cell lysis time-High power consumption - Cannot be used for extracting sensitive intracellular components.
**Chemical**	30 s–20 min	High	- Low cost and lysis time - Additional step of adding the chemical - Expensive lysis reagents
**Optical**	30 s–10 min	High	- Single cell lysis is possible - Cell specific lysis - Expensive and complex instrumentation required - Slow lysis time
**Acoustic**	3 s–1 min	Medium	- Easy integration of electrodes into microfluidic device - Fast cell lysis time - Expensive technology - Heat generation during lysis
**Electrical**	50 ms–10 min	High	- Fast lysis time - Easy integration of electrodes in device - Joule heating - Expensive equipment for lysis

## Abbreviations

PCR	Polymerase Chain Reaction
*E. coli*	*Escherichia coli*

## References

[1] Sakmann B., Neher E. (1984). Patch clamp techniques for studying ionic channels in excitable membranes. Annu. Rev. Physiol..

[2] Goodfellow M., Stackebrandt E. (1991). Nucleic Acid Techniques in Bacterial Systematics.

[3] Harrison S.T.L. (1991). Bacterial cell disruption: A key unit operation in the recovery of intracellular products. Biotechnol. Adv..

[4] Markets and Markets Cell Lysis/Cell Fractionation Market-Global Forecasts to 2021. http://www.marketsandmarkets.com/Market-Reports/cell-lysis-market260138321.html.

[5] Mark D., Haeberle S., Roth G., von Stetten F., Zengerle R. (2010). Microfluidic lab-on-a-chip platforms: Requirements, characteristics and applications. Chem. Soc. Rev..

[6] Andersson H., van den Berg A. (2003). Microfluidic devices for cellomics: A review. Sens. Actuators B Chem..

[7] Nan L., Jiang Z., Wei X. (2014). Emerging microfluidic devices for cell lysis: A review. Lab Chip.

[8] Brown R.B., Audet J. (2008). Current techniques for single-cell lysis. J. R. Soc. Interface.

[9] Mahalanabis M., Al-Muayad H., Kulinski M.D., Altman D., Klapperich C.M. (2009). Cell lysis and DNA extraction of gram-positive and gram-negative bacteria from whole blood in a disposable microfluidic chip. Lab Chip.

[10] Engler C.R. (1985). Disruption of microbial cells. Comnrehensive Biotechnoloy.

[11] Hammond S.M., Lambert P.A., Rycroft A.N. (1984). The Bacterial Cell Surface.

[12] Ghuysen J.-M. (1973). Biosynthesis of peptidoglycan. The Bacterial Membranes and Walls.

[13] McIntosh H.M. (1989). An Ultrastructural Study of Poly-/3-Hydroxybutyrate Separation from Alcaligenes Eutrophus by Selective Envelope Degradation. Ph.D. Thesis.

[14] Madigan M.T., Martinko J.M., Dunlap P.V., Clark D.P. (2008). Brock biology of microorganisms 12th edn. Int. Microbiol..

[15] Grayson P., Evilevitch A., Inamdar M.M., Purohit P.K., Gelbart W.M., Knobler C.M., Phillips R. (2006). The effect of genome length on ejection forces in bacteriophage lambda. Virology.

[16] Silhavy T.J., Kahne D., Walker S. (2010). The bacterial cell envelope. Cold Spring Harb. Perspect. Biol..

[17] Engler C.R., Robinson C.W. (1981). Disruption of candida utilis cells in high pressure flow devices. Biotechnol. Bioeng..

[18] Middelberg A.P.J. (2000). 2 microbial cell disruption by high-pressure homogenization. Downstream Processing of Proteins: Methods and Protocols.

[19] Sauer T., Robinson C.W., Glick B.R. (1989). Disruption of native and recombinant *Escherichia coli* in a high-pressure homogenizer. Biotechnol. Bioeng..

[20] Augenstein D.C., Thrasher K., Sinskey A.J., Wang D.I.C. (1974). Optimization in the recovery of a labile intracellular enzyme. Biotechnol. Bioeng..

[21] Schütte H., Kroner K.H., Hustedt H., Kula M.R. (1983). Experiences with a 20 litre industrial bead mill for the disruption of microorganisms. Enzym. Microb. Technol..

[22] Chisti Y., Moo-Young M. (1986). Disruption of microbial cells for intracellular products. Enzym. Microb. Technol..

[23] Taskova R.M., Zorn H., Krings U., Bouws H., Berger R.G. (2006). A comparison of cell wall disruption techniques for the isolation of intracellular metabolites from pleurotus and lepista sp.. Z. Naturforsch. C J. Biosci..

[24] Ho C.W., Tan W.S., Yap W.B., Ling T.C., Tey B.T. (2008). Comparative evaluation of different cell disruption methods for the release of recombinant hepatitis b core antigen from *Escherichia coli*. Biotechnol. Bioprocess Eng..

[25] Goldberg S. (2008). Mechanical/physical methods of cell disruption and tissue homogenization. Methods Mol. Biol..

[26] Johnson B.H., Hecht M.H. (1994). Cells by repeated cycles of freezing and thawing. Biotechnology.

[27] Watson J.S., Cumming R.H., Street G., Tuffnell J.M. (1987). Release of Intracellular Protein by Thermolysis.

[28] Wang B., Merva M., Williams W.V., Weiner D.B. (1995). Large-scale preparation of plasmid DNA by microwave lysis. Biotechniques.

[29] Zhu K., Jin H., He Z., Zhu Q., Wang B. (2007). A continuous method for the large-scale extraction of plasmid DNA by modified boiling lysis. Nat. Protoc..

[30] Lilly M.D., Dunnill P. (1969). Isolation of intracellular enzymes from micro-organisms-the development of a continuous process. Fermentation Advances.

[31] Balasundaram B., Harrison S., Bracewell D.G. (2009). Advances in product release strategies and impact on bioprocess design. Trends Biotechnol..

[32] Lee A.K., Lewis D.M., Ashman P.J. (2015). Microalgal cell disruption by hydrodynamic cavitation for the production of biofuels. J. Appl. Phycol..

[33] Capocellia M., Prisciandarob M., Lanciac A., Musmarraa D. (2014). Comparison between hydrodynamic and acoustic cavitation in microbial cell disruption. Chem. Eng..

[34] Fonseca L.P., Cabral J. (2002). Penicillin acylase release from *Escherichia coli* cells by mechanical cell disruption and permeabilization. J. Chem. Technol. Biotechnol..

[35] Chen Y.-C., Chen L.-A., Chen S.-J., Chang M.-C., Chen T.-L. (2004). A modified osmotic shock for periplasmic release of a recombinant creatinase from *Escherichia coli*. Biochem. Eng. J..

[36] Byreddy A.R., Gupta A., Barrow C.J., Puri M. (2015). Comparison of cell disruption methods for improving lipid extraction from thraustochytrid strains. Mar. Drugs.

[37] Bimboim H.C., Doly J. (1979). A rapid alkaline extraction procedure for screening recombinant plasmid DNA. Nucleic Acids Res..

[38] Stanbury P.F., Whitaker A. (1984). Principles of Fermentation Technology.

[39] Tamura K., Aotsuka T. (1988). Rapid isolation method of animal mitochondrial DNA by the alkaline lysis procedure. Biochem. Genet..

[40] Feliciello I., Chinali G. (1993). A modified alkaline lysis method for the preparation of highly purified plasmid DNA from *Escherichia coli*. Anal. Biochem..

[41] Sharma R., Dill B.D., Chourey K., Shah M., VerBerkmoes N.C., Hettich R.L. (2012). Coupling a detergent lysis/cleanup methodology with intact protein fractionation for enhanced proteome characterization. J. Proteome Res..

[42] PanReac AppliChem Detergents—More than Foam!. https://www.applichem.com/en/literature/brochures/brochures-biochemical-support/detergents/.

[43] Andrews B.A., Asenjo J.A. (1987). Enzymatic lysis and disruption of microbial cells. Trends Biotechnol..

[44] Salazar O., Asenjo J.A. (2007). Enzymatic lysis of microbial cells. Biotechnol. Lett..

[45] Anand H., Balasundaram B., Pandit A.B., Harrison S.T.L. (2007). The effect of chemical pretreatment combined with mechanical disruption on the extent of disruption and release of intracellular protein from *E. coli*. Biochem. Eng. J..

[46] Beebe D.J., Mensing G.A., Walker G.M. (2002). Physics and applications of microfluidics in biology. Annu. Rev. Biomed. Eng..

[47] Khandurina J., McKnight T.E., Jacobson S.C., Waters L.C., Foote R.S., Ramsey J.M. (2000). Integrated system for rapid PCR-based DNA analysis in microfluidic devices. Anal. Chem..

[48] Burns M.A., Johnson B.N., Brahmasandra S.N., Handique K., Webster J.R., Krishnan M., Sammarco T.S., Man P.M., Jones D., Heldsinger D. (1998). An integrated nanoliter DNA analysis device. Science.

[49] Lin Z., Cai Z. (2009). Cell lysis methods for high-throughput screening or miniaturized assays. Biotechnol. J..

[50] Berasaluce A., Matthys L., Mujika J., Antoñana-Díez M., Valero A., Agirregabiria M. (2015). Bead beating-based continuous flow cell lysis in a microfluidic device. RSC Adv..

[51] Pham V.T.H., Truong V.K., Mainwaring D.E., Guo Y., Baulin V.A., Al Kobaisi M., Gervinskas G., Juodkazis S., Zeng W.R., Doran P.P. (2014). Nanotopography as a trigger for the microscale, autogenous and passive lysis of erythrocytes. J. Mater. Chem. B.

[52] Di Carlo D., Jeong K.H., Lee L.P. (2003). Reagentless mechanical cell lysis by nanoscale barbs in microchannels for sample preparation. Lab Chip.

[53] Kido H., Micic M., Smith D., Zoval J., Norton J., Madou M. (2007). A novel, compact disk-like centrifugal microfluidics system for cell lysis and sample homogenization. Colloids Surf. B Biointerfaces.

[54] Kim J., Hee Jang S., Jia G., Zoval J.V., Da Silva N.A., Madou M.J. (2004). Cell lysis on a microfluidic CD (compact disc). Lab Chip.

[55] Madou M., Zoval J., Jia G., Kido H., Kim J., Kim N. (2006). Lab on a CD. Annu. Rev. Biomed. Eng..

[56] Tsougeni K., Papadakis G., Gianneli M., Grammoustianou A., Constantoudis V., Dupuy B., Petrou P.S., Kakabakos S.E., Tserepi A., Gizeli E. (2016). Plasma nanotextured polymeric lab-on-a-chip for highly efficient bacteria capture and lysis. Lab Chip.

[57] Kim J., Johnson M., Hill P., Gale B.K. (2009). Microfluidic sample preparation: Cell lysis and nucleic acid purification. Integr. Biol. Quant. Biosci. Nano Macro.

[58] Yeung S.-W., Lee T.M.-H., Cai H., Hsing I.M. (2006). A DNA biochip for on-the-spot multiplexed pathogen identification. Nucleic Acids Res..

[59] Liu R.H., Yang J., Lenigk R., Bonanno J., Grodzinski P. (2004). Self-contained, fully integrated biochip for sample preparation, polymerase chain reaction amplification, and DNA microarray detection. Anal. Chem..

[60] Buser J.R., Zhang X., Byrnes S.A., Ladd P.D., Heiniger E.K., Wheeler M.D., Bishop J.D., Englund J.A., Lutz B., Weigl B.H. (2016). A disposable chemical heater and dry enzyme preparation for lysis and extraction of DNA and RNA from microorganisms. Anal. Methods.

[61] Kashyap A., Autebert J., Delamarche E., Kaigala G.V. (2016). Selective local lysis and sampling of live cells for nucleic acid analysis using a microfluidic probe. Sci. Rep..

[62] Hall J.A., Felnagle E., Fries M., Spearing S., Monaco L., Steele A. (2006). Evaluation of cell lysis procedures and use of a micro fluidic system for an automated DNA-based cell identification in interplanetary missions. Planet. Space Sci..

[63] Kim Y.-B., Park J.-H., Chang W.-J., Koo Y.-M., Kim E.-K., Kim J.-H. (2006). Statistical optimization of the lysis agents for gram-negative bacterial cells in a microfluidic device. Biotechnol. Bioprocess Eng..

[64] Heo J., Thomas K.J., Seong G.H., Crooks R.M. (2003). A microfluidic bioreactor based on hydrogel-entrapped *E. coli*: Cell viability, lysis, and intracellular enzyme reactions. Anal. Chem..

[65] Sethu P., Anahtar M., Moldawer L.L., Tompkins R.G., Toner M. (2004). Continuous flow microfluidic device for rapid erythrocyte lysis. Anal. Chem..

[66] Schilling E.A., Kamholz A.E., Yager P. (2002). Cell lysis and protein extraction in a microfluidic device with detection by a fluorogenic enzyme assay. Anal. Chem..

[67] Marc P.J., Sims C.E., Allbritton N.L. (2007). Coaxial-flow system for chemical cytometry. Anal. Chem..

[68] Ocvirk G., Salimi-Moosavi H., Szarka R.J., Arriaga E.A., Andersson P.E., Smith R., Dovichi N.J., Harrison D.J. (2004). B-galactosidase assays of single-cell lysates on a microchip: A complementary method for enzymatic analysis of single cells. Proc. IEEE.

[69] Abolmaaty A., El-Shemy M.G., Khallaf M.F., Levin R.E. (1998). Effect of lysing methods and their variables on the yield of *Escherichia coli* O157: H7 DNA and its PCR amplification. J. Microbiol. Methods.

[70] Huang S.-H., Hung L.-Y., Lee G.-B. (2016). Continuous nucleus extraction by optically-induced cell lysis on a batch-type microfluidic platform. Lab Chip.

[71] Kremer C., Witte C., Neale S.L., Reboud J., Barrett M.P., Cooper J.M. (2014). Shape-dependent optoelectronic cell lysis. Angew. Chem..

[72] Rau K.R., Quinto-Su P.A., Hellman A.N., Venugopalan V. (2006). Pulsed laser microbeam-induced cell lysis: Time-resolved imaging and analysis of hydrodynamic effects. Biophys. J..

[73] Li H., Sims C.E., Wu H.Y., Allbritton N.L. (2001). Spatial control of cellular measurements with the laser micropipet. Anal. Chem..

[74] Hellman A.N., Rau K.R., Yoon H.H., Venugopalan V. (2008). Biophysical response to pulsed laser microbeam-lnduced cell lysis and molecular delivery. J. Biophotonics.

[75] Quinto-Su P.A., Lai H.-H., Yoon H.H., Sims C.E., Allbritton N.L., Venugopalan V. (2008). Examination of laser microbeam cell lysis in a PDMS microfluidic channel using time-resolved imaging. Lab Chip.

[76] Wan W., Yeow J.T. (2011). Study of a novel cell lysis method with titanium dioxide for lab-on-a-chip devices. Biomed. Microdevices.

[77] Taller D., Richards K., Slouka Z., Senapati S., Hill R., Go D.B., Chang H.-C. (2015). On-chip surface acoustic wave lysis and ion-exchange nanomembrane detection of exosomal RNA for pancreatic cancer study and diagnosis. Lab Chip.

[78] Chen Y., Ding X., Steven Lin S.-C., Yang S., Huang P.-H., Nama N., Zhao Y., Nawaz A.A., Guo F., Wang W. (2013). Tunable nanowire patterning using standing surface acoustic waves. ACS Nano.

[79] Guo F., Li P., French J.B., Mao Z., Zhao H., Li S., Nama N., Fick J.R., Benkovic S.J., Huang T.J. (2015). Controlling cell–cell interactions using surface acoustic waves. Proc. Natl. Acad. Sci. USA.

[80] Marentis T.C., Kusler B., Yaralioglu G.G., Liu S., Haeggstrom E.O., Khuri-Yakub B.T. (2005). Microfluidic sonicator for real-time disruption of eukaryotic cells and bacterial spores for DNA analysis. Ultrasound Med. Biol..

[81] Taylor M.T., Belgrader P., Furman B.J., Pourahmadi F., Kovacs G.T., Northrup M.A. (2001). Lysing bacterial spores by sonication through a flexible interface in a microfluidic system. Anal. Chem..

[82] Reboud J., Bourquin Y., Wilson R., Pall G.S., Jiwaji M., Pitt A.R., Graham A., Waters A.P., Cooper J.M. (2012). Shaping acoustic fields as a toolset for microfluidic manipulations in diagnostic technologies. Proc. Natl. Acad. Sci. USA.

[83] Wei X., Nan L., Ren J., Liu X., Jiang Z. Surface acoustic wave induced thermal lysis of red blood cells in microfluidic channel. Proceedings of the 19th International Conference on Miniaturized Systems for Chemistry and Life Sciences.

[84] Tandiono T., Ow D.S., Driessen L., Chin C.S., Klaseboer E., Choo A.B., Ohl S.W., Ohl C.D. (2012). Sonolysis of *Escherichia coli* and pichia pastoris in microfluidics. Lab Chip.

[85] Zhang H., Jin W. (2004). Determination of different forms of human interferon-gamma in single natural killer cells by capillary electrophoresis with on-capillary immunoreaction and laser-induced fluorescence detection. Electrophoresis.

[86] Ohshima T., Sato M., Saito M. (1995). Selective release of intracellular protein using pulsed electric field. J. Electrost..

[87] Ameri S.K., Singh P.K., Dokmeci M.R., Khademhosseini A., Xu Q., Sonkusale S.R. (2014). All electronic approach for high-throughput cell trapping and lysis with electrical impedance monitoring. Biosens. Bioelectron..

[88] Jiang F., Chen J., Yu J. (2016). Design and application of a microfluidic cell lysis microelectrode chip. Instrum. Sci. Technol..

[89] De Lange N., Tran T.M., Abate A.R. (2016). Electrical lysis of cells for detergent-free droplet assays. Biomicrofluidics.

[90] Escobedo C., Bürgel S.C., Kemmerling S., Sauter N., Braun T., Hierlemann A. (2015). On-chip lysis of mammalian cells through a handheld corona device. Lab Chip.

[91] Besant J.D., Das J., Sargent E.H., Kelley S.O. (2013). Proximal bacterial lysis and detection in nanoliter wells using electrochemistry. ACS Nano.

[92] Gabardo C.M., Kwong A.M., Soleymani L. (2015). Rapidly prototyped multi-scale electrodes to minimize the voltage requirements for bacterial cell lysis. Analyst.

[93] Li X., Zhao S., Hu H., Liu Y.-M. (2016). A microchip electrophoresis-mass spectrometric platform with double cell lysis nano-electrodes for automated single cell analysis. J. Chromatogr. A.

[94] Wassermann K.J., Maier T., Keplinger F., Peham J.R., Jarm T., Kramar P. (2016). A novel sample preparation concept for sepsis diagnostics using high frequency electric fields. Proceedings of the 1st World Congress on Electroporation and Pulsed Electric Fields in Biology, Medicine and Food & Environmental Technologies.

[95] Ma S., Bryson B.D., Sun C., Fortune S.M., Lu C. (2016). RNA extraction from a mycobacterium under ultrahigh electric field intensity in a microfluidic device. Anal. Chem..

[96] Islam M.I., Kuryllo K., Selvaganapathy P.R., Li Y., Deen M.J. A microfluidic sample preparation device for pre-concentration and cell lysis using a nanoporous membrane. Proceedings of the 17th International Conference on Miniaturized Systems for Chemistry and Life Sciences.

[97] Sedgwick H., Caron F., Monaghan P.B., Kolch W., Cooper J.M. (2008). Lab-on-a-chip technologies for proteomic analysis from isolated cells. J. R. Soc. Interface.

[98] Lim J.K., Zhou H., Tilton R.D. (2009). Liposome rupture and contents release over coplanar microelectrode arrays. J. Colloid Interface Sci..

[99] Lu K.Y., Wo A.M., Lo Y.J., Chen K.C., Lin C.M., Yang C.R. (2006). Three dimensional electrode array for cell lysis via electroporation. Biosens. Bioelectron..

[100] Church C., Zhu J., Huang G., Tzeng T.-R., Xuan X. (2010). Integrated electrical concentration and lysis of cells in a microfluidic chip. Biomicrofluidics.

[101] Lee S.W., Yowanto H., Tai Y.C. A micro cell lysis device. Proceedings of the IEEE Eleventh Annual International Workshop on Micro Electro Mechanical Systems (MEMS 98). An Investigation of Micro Structures, Sensors, Actuators, Machines and Systems (Cat. No. 98CH36176).

[102] Lee D.W., Cho Y.-H. (2007). A continuous electrical cell lysis device using a low dc voltage for a cell transport and rupture. Sens. Actuators B Chem..

[103] Wang H.Y., Lu C. (2006). Electroporation of mammalian cells in a microfluidic channel with geometric variation. Anal. Chem..

[104] Lu H., Schmidt M.A., Jensen K.F. (2005). A microfluidic electroporation device for cell lysis. Lab Chip.

[105] De la Rosa C., Kaler K.V. (2006). Electro-disruption of *Escherichia coli* bacterial cells on a microfabricated chip. Conf. Proc. IEEE Eng. Med. Biol. Soc..

[106] Wang H.Y., Lu C. (2006). Microfluidic chemical cytometry based on modulation of local field strength. Chem. Commun..

[107] Wang H.Y., Bhunia A.K., Lu C. (2006). A microfluidic flow-through device for high throughput electrical lysis of bacterial cells based on continuous dc voltage. Biosens. Bioelectron..

[108] Bao N., Lu C. (2008). A microfluidic device for physical trapping and electrical lysis of bacterial cells. Appl. Phys. Lett..

[109] Svec D., Andersson D., Pekny M., Sjöback R., Kubista M., Ståhlberg A. (2013). Direct cell lysis for single-cell gene expression profiling. Front. Oncol..

[110] Kemmerling S., Arnold S.A., Bircher B.A., Sauter N., Escobedo C., Dernick G., Hierlemann A., Stahlberg H., Braun T. (2013). Single-cell lysis for visual analysis by electron microscopy. J. Struct. Biol..

